# Supplementary material for: Nebulized fusion inhibitory peptide protects cynomolgus macaques from measles virus infection
Source: Nat Commun. 2022 Oct 28;13:6439. doi: 10.1038/s41467-022-33832-6 (PMC9616412; doi:10.1038/s41467-022-33832-6)
Supplement: Supplementary file 1 — Supplementary Information [file 41467_2022_33832_MOESM1_ESM.pdf]

Supplementary Materials

Nebulized fusion inhibitory peptide protects cynomolgus macaques from measles virus infection

Olivier Reynard[1], Claudia Gonzalez[1], Claire Dumont[1], Mathieu Iampietro[1], Marion Ferren[1], Sandrine Le Guellec[2], Lajoie Laurie[3], Cyrille Mathieu[1], Gabrielle Carpentier[4], Georges Roseau[4], Francesca T. Bovier[6], Yun Zhu[6,7], Deborah Le Pennec[5], Jérome Montharu[4], Amin Addetia[8], Alexander L. Greninger[8], Christopher A. Alabi[9], Elise Brisebard[10], Anne Moscona[6,11,12], Laurent Vecellio[4], Matteo Porotto[6,13], Branka Horvat[1]

- 2 supplementary tables

- 8 Supplementary figures

Table S1. Performance of nebulizers used in the study, loaded with 3 ml of either saline or peptide.

Table S2 Deposition of the aerosol in organs of nebulized animals

Fig. S1. Evaluation of HRC4 dose and treatment schedule in CD150xIFNα/β R KO murine model of MeV infection.

Fig. S2. Distribution in nebulized HRC4 peptide in lungs of cynomolgus monkeys.

Fig. S3. Histopathological analysis of lungs from HRC4-nebulised cynomolgus macaques**.**

Fig. S4. Analysis of the IgE production in serum and eosinophil infiltration in the lungs following HRC4 nebulization of macaques.

Fig. S5. Schematic presentation of the peptide and virus deposition in the cell culture model.

Fig. S5. Evolution of the hematological parameters during the MeV infection**.**

Fig. S7. Evolution of major PBMC populations in blood of cynomolgus macaques.

Fig. S8. Gating strategies used in cytofluorometry analysis in figures 6, 7, 8 and S7.

Supplementary Table S1. Performance of nebulizers used in the study, loaded with 3 ml of either saline or peptide and presented as mean ± SD of four tested devices.

	[1]VMD (μm)	[2]< 5μm (%)	[2]< 2μm (%)	[3]Duration (min)	[4]Output rate (ml/min)	[5]Residual volume (ml)	[6]Output (%)
NaCl (0.9 %)	4.37 +/- 0.16	59.8 +/- 2,8	8.5 +/- 0.8	6.9 +/- 1.9	0.46 +/- 0.13	0.05 +/- 0.01	98.5 +/- 0.4
HRC4 (4 mg/ml)	4.43 +/- 0.26	57.4 +/- 3.9	13.9 +/- 2.0	9.3 +/- 1.2	0.32 +/- 0.04	0.06 +/- 0.04	98.1 +/- 1.3

[1] VMD for Volume Median Diameter, presents the mean size of generated aerosols, measured by laser diffraction method (Spraytec Malvern Instrument)

[2] Percentage of particles smaller than 5 μm or 2 μm, presents the fraction of aerosol below the indicated size, corresponding to the aerosol penetrating into either lungs in general (< 5μm) or into alveolar regions of lungs (< 2μm).

[3] Duration corresponds to the time necessary to achieve complete aerosolization of the 3 ml loaded solution

[4] Output rate presents the aerosol flow rate in ml per minute

[5] Residual volume corresponds to the volume of liquid remaining in the reservoir at the end of the nebulization

[6] Output corresponds to the percentage of liquid volume delivered by the nebulizer compared to the initial charge of each nebulizer (n = 4).

Supplementary Table S2. Deposition of the aerosol in organs of nebulized animals.

***Deposition in macaque (%)**	**Animal 1.1**	**Animal 1.2**	**Animal 2.1**	**Animal 2.2**	**Mean +/- SD**
Lungs	8.0	11.3	15.5	10.7	11.4 +/- 3.1
Upper respiratory Tract	15.5	28.0	44.4	30.3	29.5 +/- 11.9
Stomach	15.7	22.0	0.4	9.7	11.9 +/-9.2
Esophagus / trachea	1.2	1.7	1.1	9.7	3.4 +/- 4.2

* ^{99m}TC-DTPA (74 MBq) was administrated in 3 ml NaCl 0.9% by prototype mesh nebulizer in four experiments (2 different animals were nebulized independently twice). The deposition was analyzed by E-cam gamma camera and calculated from the generated digitalized images.

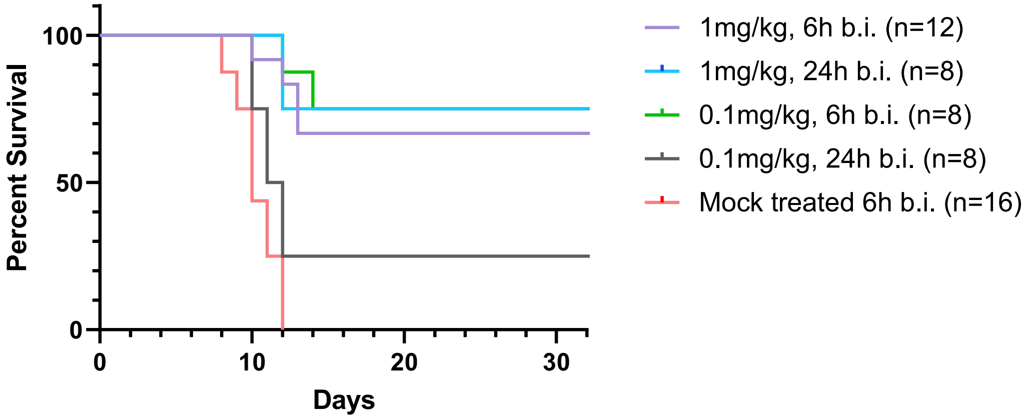

Supplementary Figure S1. Evaluation of HRC4 dose and treatment schedule in CD150xIFNα/βR KO murine model of MeV infection. Mice were pretreated intranasally either 6 h or 24 h before infection (b.i.) with the indicated dose of HRC4 and inoculated with 10^4 PFU of MeV IC323. HRC4 lipopeptide at 1 mg/kg efficiently protected CD150xIFNα/βR KO mice from intranasal MeV infection when given 6 h and 24 h before infection ($p < 0.0001$, Mantel-Cox test)

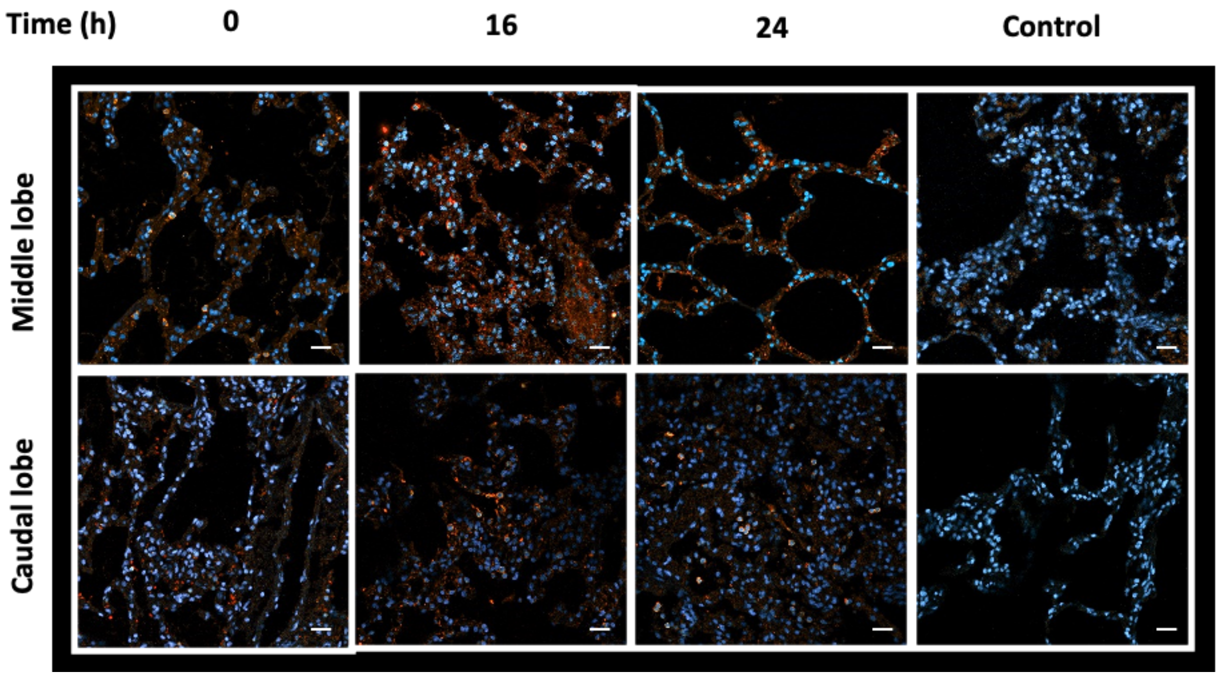

Supplementary figure S2. Distribution in nebulized HRC4 peptide in lungs of cynomolgus monkeys. HRC4 nebulization was made at indicated time points: 15 min (T0), 16 h (T16) or 24 h (T24) under mechanical ventilation prior to euthanasia and control NHP did not receive peptide. Peptide biodistribution was assessed in caudal (low), middle (medium) and cranial lobes (upper) (Fig. 3B), of the right lung on 5 slides prepared from each section. Paraffin embedded lung sections from NHPs were analyzed by immunofluorescence, using rabbit anti-HRC4 peptide and goat anti-rabbit Alexa 555 (orange staining) and DAPI was used to stain nuclei (blue staining), and imaging was done using a Zeiss LSM800 confocal microscope at 20x (scale bars: 20 µm).

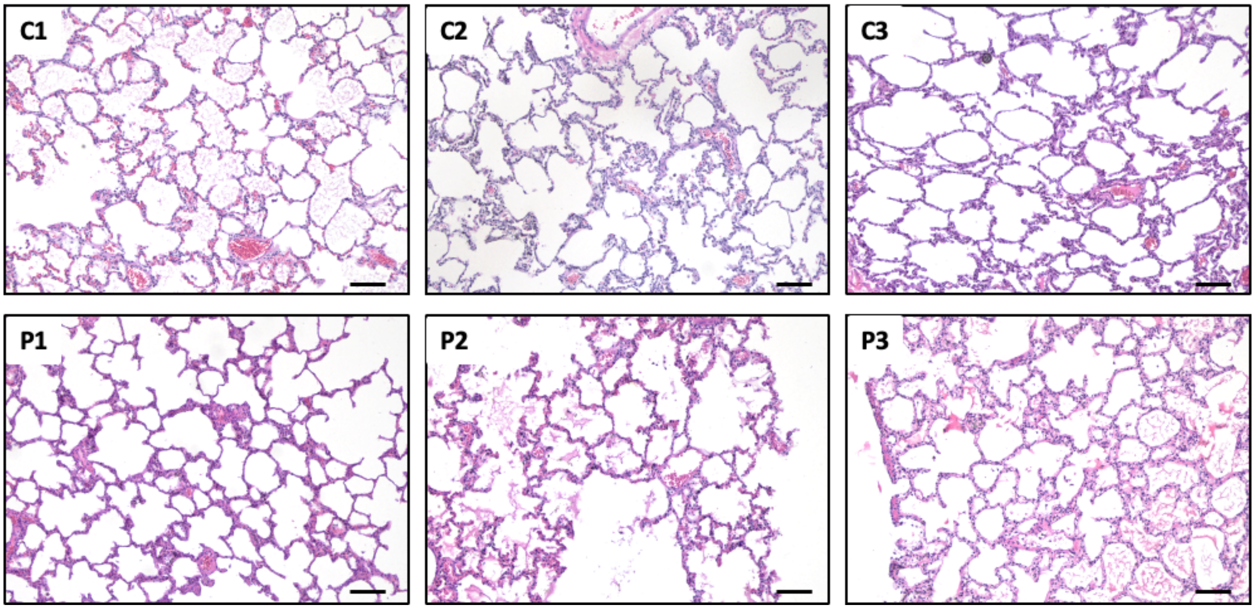

Supplementary figure S3. Histopathological analysis of lungs from HRC4-nebulised cynomolgus macaques. Animals were nebulized 15 min (C1, C2, C3), 16 h (P2) or 24 h (P1, P3) prior to euthanasia and lung sections from paraffin-embedded organs from nebulized animals were stained with hematoxylin and eosin. Analysis of 3 lung sections originating from distinct lobes from each animal presented and absence of any visible adverse effects**.** Images were taken on a Nikon Eclipse Ts2R microscope at 10x and scale bars correspond to 200 µm.

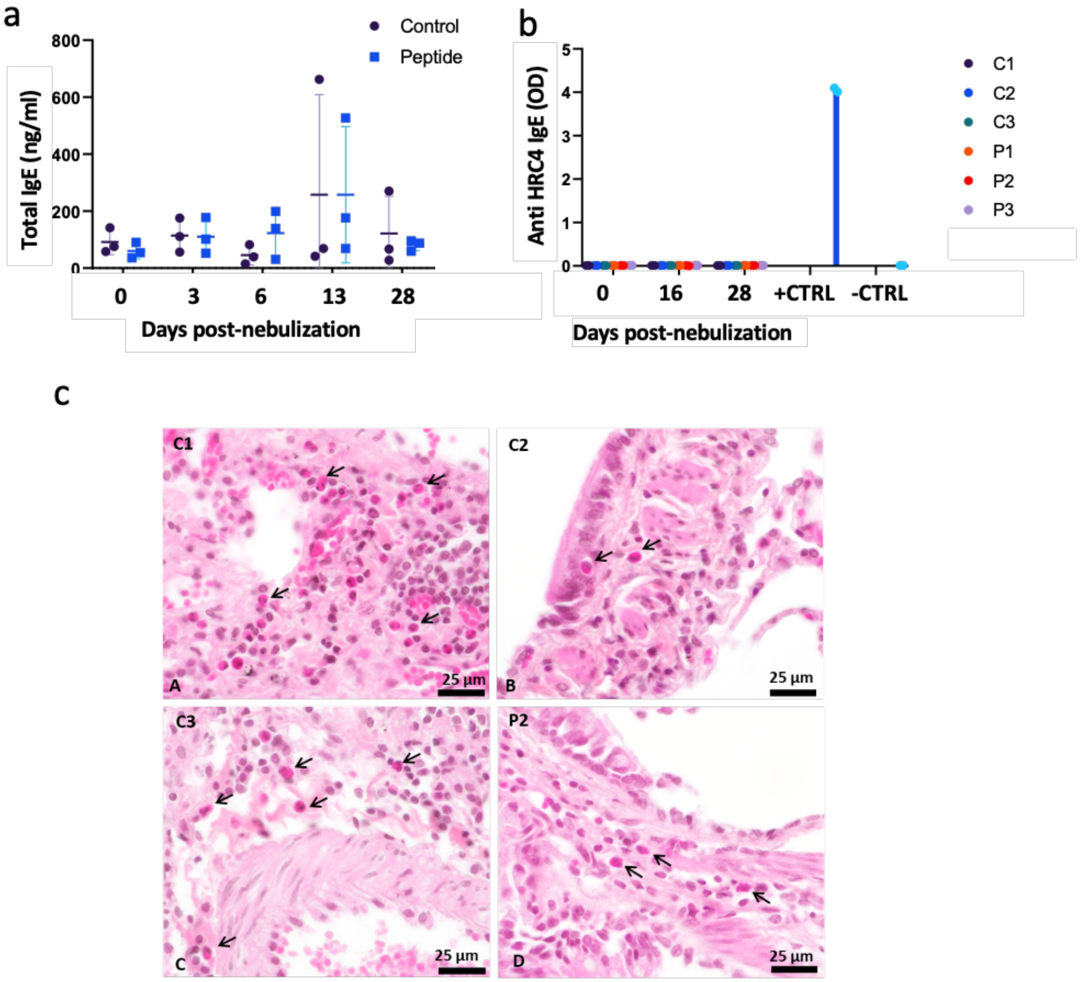

Supplementary figure S4. Analysis of the IgE production in serum and eosinophil infiltration in the lungs following HRC4 nebulization of macaques. (**a**) Total IgE was measured in sera by ELISA following either saline (control) or peptide nebulization, (n=3 for both groups) (two tailed Mann-Whitney test, p=0,99) Data are presented as mean values $\pm$ SD. (**b**) Determination of HRC-4 specific IgE by ELISA in the serum of macaques nebulized with either saline (control, C) or peptide (P). Positive control (CTRL) was a rabbit IgG anti-HRC4 detected with an anti-rabbit Ab coupled to HRP, while in negative control only secondary Ab was added. Results were reproduced in 2 independent experiments and data are presented as mean values $\pm$ SD. Source data are provided as a Source Data file. (**c**) Lung sections from paraffin-embedded organs from nebulized macaques were stained with hematoxylin-eosin-saffron. In 4 animals, C1, 2 and 3 and P2, a minimal but noticeable lung eosinophil infiltration was observed, while no eosinophil infiltration was visible in lungs from P1 and P3 macaques. Representative images of those 4 animals are presented here. For the six animals, six lung sections were analyzed by a pathologist and corresponding to right and left lungs and cranial, middle and caudal lobes. Some eosinophils are indicated by arrows. Images were taken on a Nikon Eclipse Ci microscope equipped with a Nikon DS-Fi3 camera and with a Nikon Apo Lambda 40x objective.

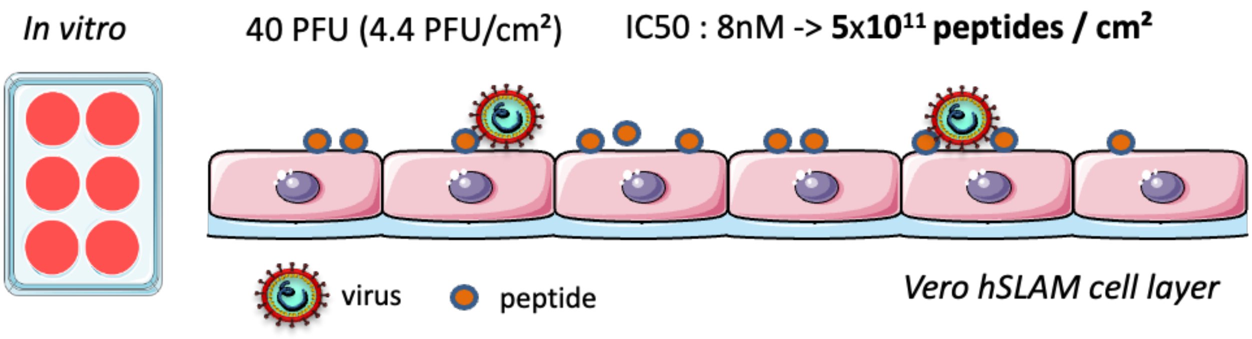

Supplementary figure S5. **Schematic presentation of the peptide and virus deposition in the cell culture model.** Peptide and virus deposition in the *in vitro* assays, presented in the Fig. 2c and d, performed in 6 well plates (9 cm^2/well), using 40 PFU of MeV/well, was calculated giving a ratio of 4.4 PFU/cm^2. Dose response experiments indicated an IC_{50} of 8 nM, which correspond to a total of $4.81x10^{12}$ molecules of peptides per well and a ratio to surface of $5x10^{11}$ peptides/cm^2, presenting the amount of peptide very close to the one deposited in macaques' lung (Fig. 5).

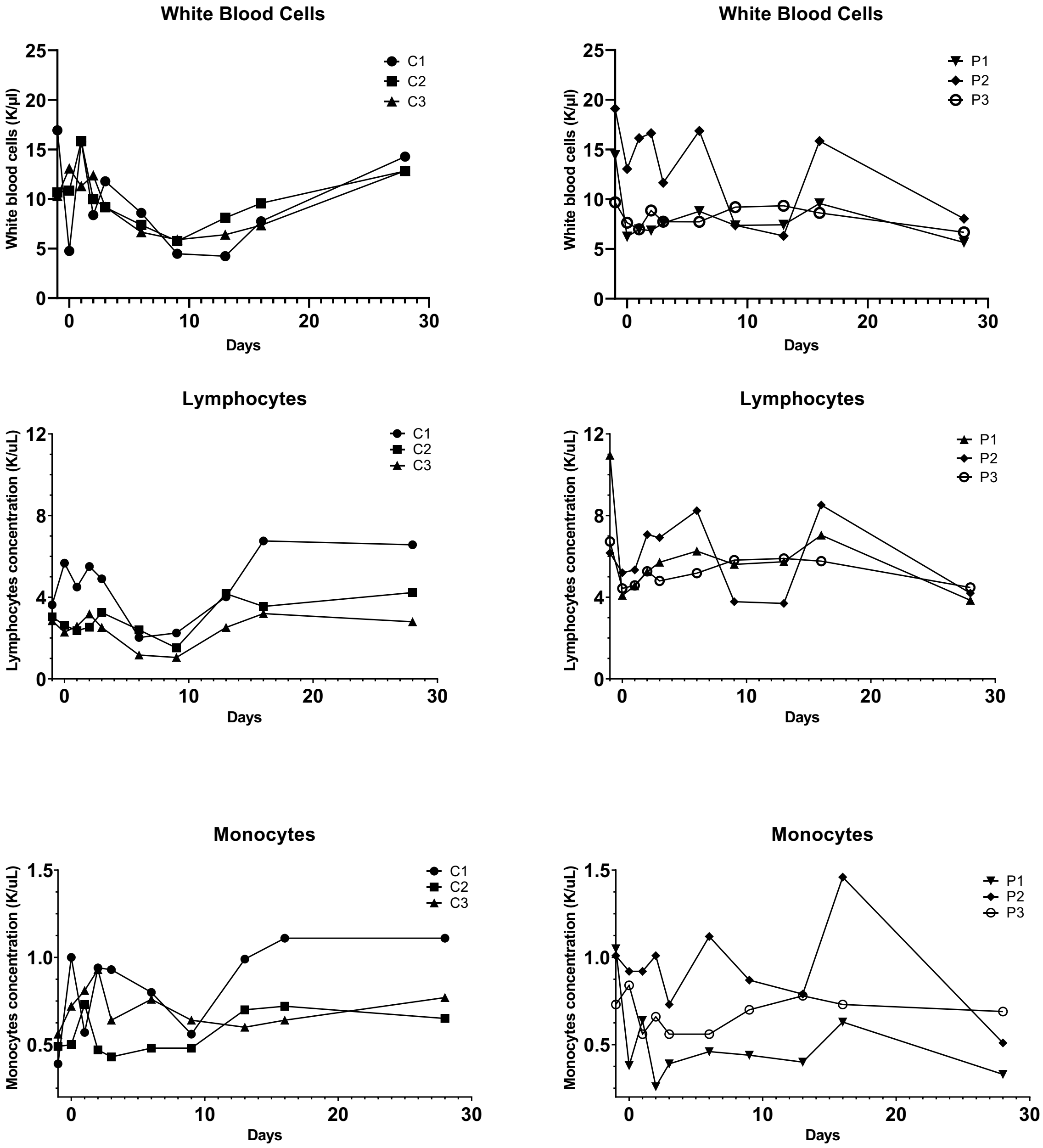

Supplementary figure S6. **Evolution of the hematological parameters during the MeV infection.** Major PBMC populations in blood of cynomolgus macaques were followed using Sysmex XT2000i Vet automatic analyzer, after nebulization of either 3 ml of 0.9% NaCl (C) or 4 mg/kg of HRC4 peptide (P) and MeV infection. Transient lymphopenia is observed in the absence of peptide protection.

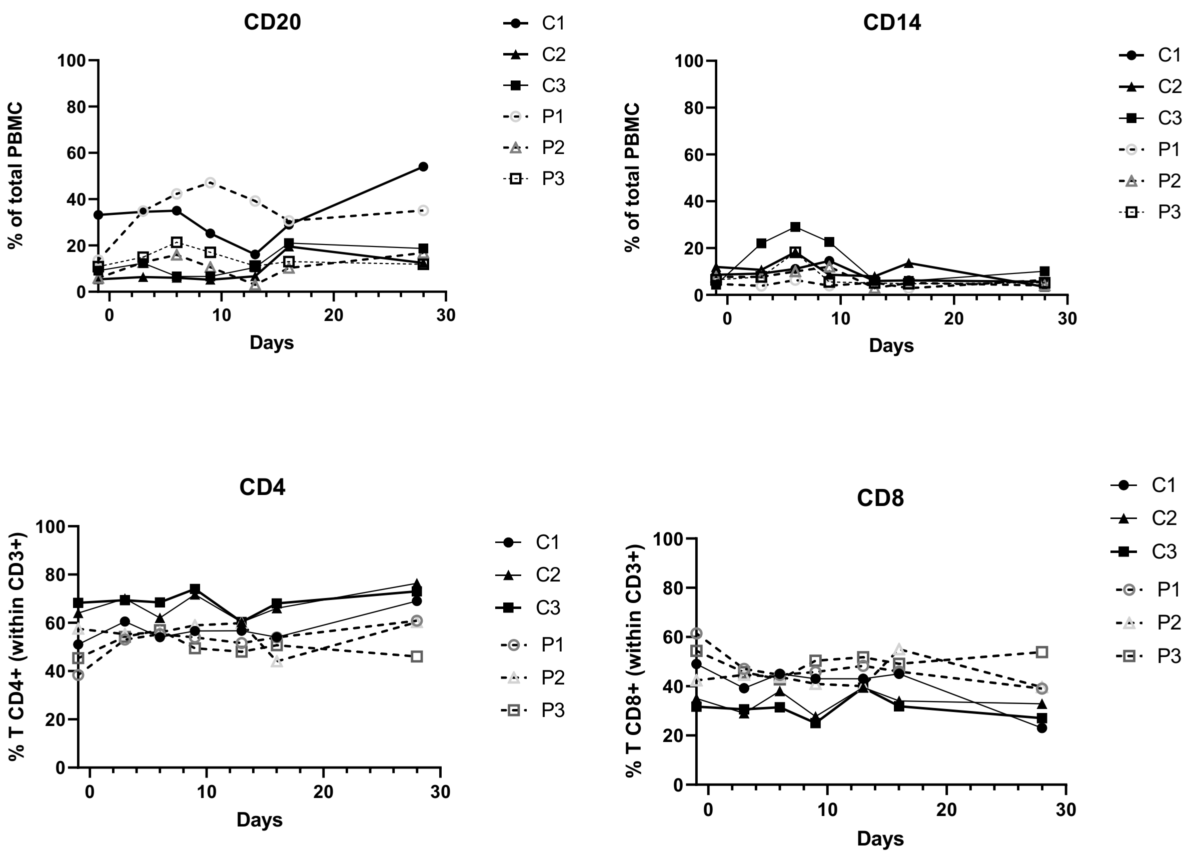

Supplementary figure S7. **Evolution of major PBMC populations in blood of cynomolgus macaques.** Following nebulization of either 0.9% NaCl (C) or 4 mg/kg of HRC4 peptide (P) and MeV infection, indicated cell populations were followed in the blood of animals at indicated time points, using a MACSQuant® 10 flow cytometer (Miltenyi).

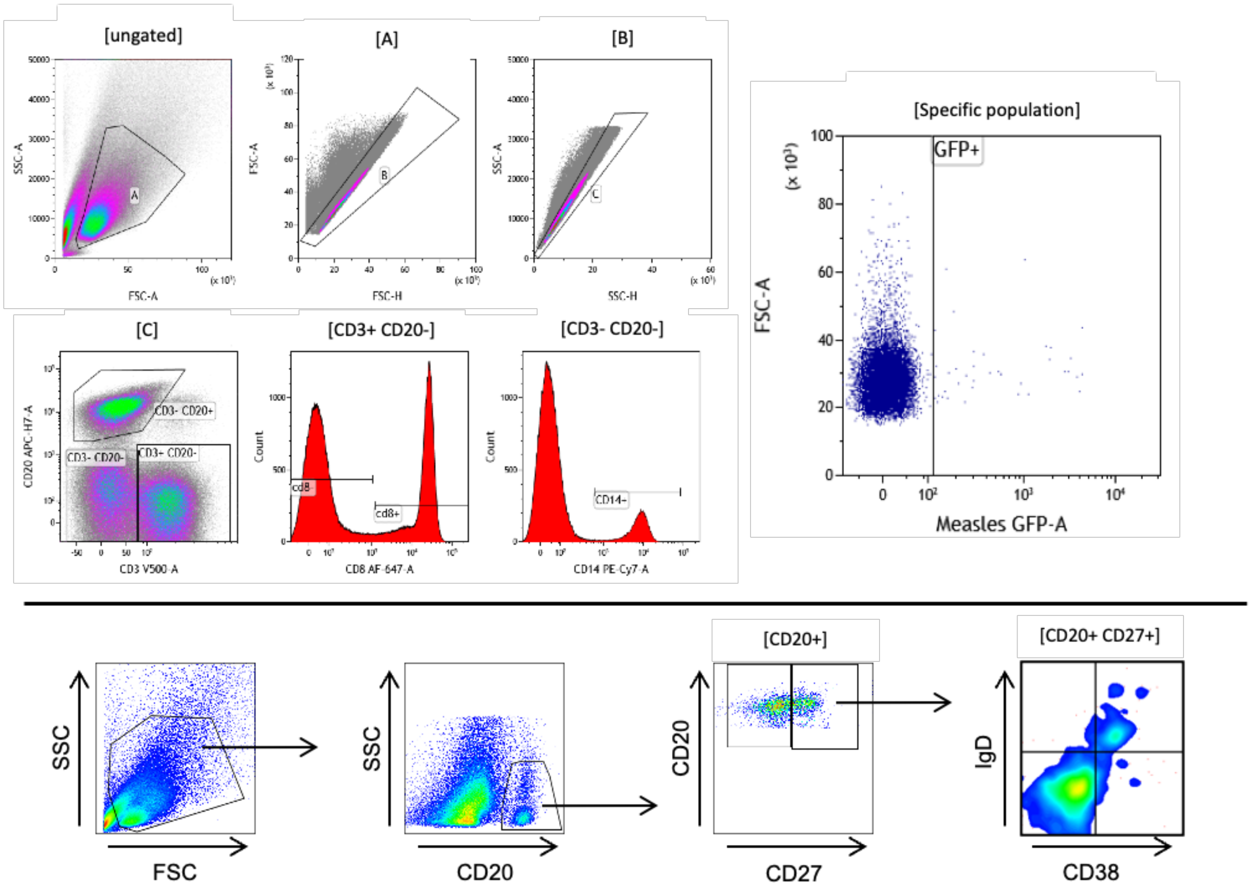

Supplementary figure S8. Gating strategies used in cytofluorometry analysis. Top panel displays the gating strategy to analyze GFP expression in specific cell population (B, lymphocytes, CD8$^{+/-}$ T lymphocytes and monocytes). Lower panel presents the characterization of B lymphocytes subpopulations to discriminate class switched and unswitched immunoglobulin secreting B cells. In figures 6 and 7 and supplementary S7 cells were first gated for morphology (FSC-A/SSC-A), then for duplet exclusion (FSC-A/FSC-H, then SSC-A/SSC-H). B cell population was determined as CD3$^-$ CD20$^+$ cells and percentage of GFP positive B cells was analyzed on a FSC-A /GFP dot plot. Monocytes infection was determined on a FSC-A/GFP dot plot gated on CD3$^-$CD20$^-$. T cells subpopulations infection were determined on FSC-A/GFP dot plot gated on CD20$^-$CD3$^+$CD8$^-$ or CD20$^-$CD3$^+$CD8$^+$ cells. In figure 8 cells were first gated for morphology (FSC-A/SSC-A) and then for CD20$^+$/CD27+. Finally, a dot plot CD38 vs IgD gated on CD20$^+$/CD27$^+$ cells was used to quantify CD38$^+$/IgD$^+$ and CD38$^+$/IgD$^-$ cells.
