# Supplementary material for: Nebulized fusion inhibitory peptide protects cynomolgus macaques from measles virus infection
Source: Nat Commun. 2022 Oct 28;13:6439. doi: 10.1038/s41467-022-33832-6 (PMC9616412; doi:10.1038/s41467-022-33832-6)
Supplement: Supplementary file 3 — Reporting Summary [file 41467_2022_33832_MOESM3_ESM.pdf]

nature research

Corresponding author(s): Branka Horvat

Last updated by author(s): 2022/09/16

Reporting Summary

Nature Research wishes to improve the reproducibility of the work that we publish. This form provides structure for consistency and transparency in reporting. For further information on Nature Research policies, see our Editorial Policies and the Editorial Policy Checklist.

Statistics

For all statistical analyses, confirm that the following items are present in the figure legend, table legend, main text, or Methods section.

n/a	Confirmed	
☐	☒	The exact sample size (*n*) for each experimental group/condition, given as a discrete number and unit of measurement
☐	☒	A statement on whether measurements were taken from distinct samples or whether the same sample was measured repeatedly
☐	☒	The statistical test(s) used AND whether they are one- or two-sided<br>*Only common tests should be described solely by name; describe more complex techniques in the Methods section.*
☒	☐	A description of all covariates tested
☐	☒	A description of any assumptions or corrections, such as tests of normality and adjustment for multiple comparisons
☐	☒	A full description of the statistical parameters including central tendency (e.g. means) or other basic estimates (e.g. regression coefficient) AND variation (e.g. standard deviation) or associated estimates of uncertainty (e.g. confidence intervals)
☐	☒	For null hypothesis testing, the test statistic (e.g. *F*, *t*, *r*) with confidence intervals, effect sizes, degrees of freedom and *P* value noted<br>*Give P values as exact values whenever suitable.*
☒	☐	For Bayesian analysis, information on the choice of priors and Markov chain Monte Carlo settings
☒	☐	For hierarchical and complex designs, identification of the appropriate level for tests and full reporting of outcomes
☒	☐	Estimates of effect sizes (e.g. Cohen's *d*, Pearson's *r*), indicating how they were calculated

Our web collection on statistics for biologists contains articles on many of the points above.

Software and code

Policy information about availability of computer code

Data collection

MACSQuant®10 flow cytometer (Miltenyi).
Zeiss LSM800 confocal microscope
Spraytec™ instrument (Malvern Instruments Ltd., Malvern, UK)
Spraytec inhalation cell (Malvern Instruments Ltd., Malvern, UK)
StepOnePlus Real-Time PCR System Applied Biosystems and StepOnePlus v2.3 Thermo Fisher Scientific https://www.thermofisher
Tecan M1000PRO / Infinite 200 Pro Lifesciences, TecanTM https://lifesciences.tecan.com/plate_readers/infinite_200_pro
Multiskan FC (Thermo)
Orbiter 75 Ecam, Siemens healthcare
Radical-7® Pulse CO-Oximeter (Masimo)
Dräger Primus anesthesia machine.
Hematometer Procyte DX (IDEXX)
Konelab 30 (ThermoFisher).
FastGene Blue/Green LED Flashlight (Nippon genetics).

Data analysis

GraphPad Prism 8.3.0 GraphPad Software Inc. https://www.graphpad.com/scientific-software/prism
ImageJ 1.52p FiJi package Image J https://imagej.net/Fiji
Analysis of RT-qPCR results was done by absolute quantification using a standard range (Steponeplus V2.3 Thermo).
FlowJo 10.8 (Becton Dickinson) and Kaluza 2.1 (Beckman Coulter) flow cytometry software

For manuscripts utilizing custom algorithms or software that are central to the research but not yet described in published literature, software must be made available to editors and reviewers. We strongly encourage code deposition in a community repository (e.g. GitHub). See the Nature Research guidelines for submitting code & software for further information.

April 2020

Data

Policy information about availability of data

All manuscripts must include a data availability statement. This statement should provide the following information, where applicable:

- Accession codes, unique identifiers, or web links for publicly available datasets
- A list of figures that have associated raw data
- A description of any restrictions on data availability

The authors declare that the data supporting the findings of this study is available from the corresponding author upon request. All relevant data including the numerical and statistical source data that underlie the graphs in figures, are provided with the paper and extended data.

Field-specific reporting

Please select the one below that is the best fit for your research. If you are not sure, read the appropriate sections before making your selection.

☒ Life sciences ☐ Behavioural & social sciences ☐ Ecological, evolutionary & environmental sciences

For a reference copy of the document with all sections, see nature.com/documents/nr-reporting-summary-flat.pdf

Life sciences study design

All studies must disclose on these points even when the disclosure is negative.

Sample size	Sample size for the experiment with cynomolgus monkeys was determined by the availlability of monkeys not vaccinated against mealses (extremely rare currently) and in respect with the ethical criteria to use the minimal number of animals in each experiment. Equal numbers of animals were assigned to treated and non treated groups. Sample group size for murine experiments was chosen based on previous results on survival after a lethal chellenge with measles virus.
Data exclusions	Data were not excluded.
Replication	All data reported in this study was reproduced in at least two independent biological replicates, as well as (when possible) via alternative independent methods.
Randomization	For the experiements with nonhuman primates, animals were distributed in each groups based on their weight, aiming to obtain an equal distribution between the groups. For the murine experiments samples were assigned to different groups randomly.
Blinding	Blinding of investigators to group allocation was performed for immunofluorescence analysis and during sample collection and preparation (PBMC, serum, RNA). Blinding was not performed in experiments involving monkeys due the experimental design and the potential presence of highly contagious shedded measles virus. Indeed, it was mandatory to perform experiments in surgical room on treated non-contagious monkeys first in order to avoid potential contamination by released aerosolized viral particles from non-treated animals. However, in experiments involving mice, where shedding of the virus has never been documented, the blinding was regularly performed. However, in experiments with mice, where shedding of the virus has never been observed, the blinding was regulary performed.

Reporting for specific materials, systems and methods

We require information from authors about some types of materials, experimental systems and methods used in many studies. Here, indicate whether each material, system or method listed is relevant to your study. If you are not sure if a list item applies to your research, read the appropriate section before selecting a response.

Materials & experimental systems

n/a	Involved in the study	
☐	☒	Antibodies
☐	☒	Eukaryotic cell lines
☒	☐	Palaeontology and archaeology
☐	☒	Animals and other organisms
☒	☐	Human research participants
☒	☐	Clinical data
☒	☐	Dual use research of concern

Methods

n/a	Involved in the study	
☒	☐	ChIP-seq
☐	☒	Flow cytometry
☒	☐	MRI-based neuroimaging

Antibodies

Antibodies used	Alexa 555 conjugated donkey anti-rabbit InvitrogenTM Cat# A31572 (dilution 1/500 as suggested on https://ww.thermofisher.com)<br>CD150 BV421 clone A12 (BD Bioscience cat# 562875) (4µl per test)<br>CD3 V500 clone SP34-2 (BD Bioscience cat# 560770) (4µl per test)

CD8 Alexa647 clone RPA-T8 (BD Bioscience cat#557708) (4µl per test)
CD14 PECy7 clone M5E2 (BD Bioscience cat#561385) (4µl per test)
CD20 APCH7 clone 2H7 , (BD Bioscience cat#560734) (4µl per test)
TCRaV7.2 PE clone3C10 (Biolegend cat#351706) (4µl per test)
CCR7 PECy7clone G043H7(Biolegend cat#353226) (4µl per test)
CD45RA APCH7 clone 5H9 (BD Bioscience cat#561212) (4µl per test)
CD38 PECy7 clone HB8 (BD Bioscience cat#335825) (4µl per test)
IgD BV510 clone IA6-2 (BD Bioscience cat#563034) (4µl per test)
cd27 Alexa647one O323 (Biolegend cat#302812) (4µl per test)
All above Biolegend and BD bioscience antibodies were recommended to be used at 5µl per 100µl samples , we internally validated that 4µl was sufficient for proper staining of our samples.
Goat anti-monkey IgG-A-M HRP (Sigma Aldrich) (1/30 000) https://www.sigmaaldrich.com/FR/fr/specification-sheet/SIGMA/SAB3700770
Rabbit anti monkey IgE - biotin labeled (Gentaur-alpha diagnostic) (1/2000, recommended 1/1000-1/10 000° for ELISA on manufacturer datasheet)
Streptavidin-HRP (RnD system) (1/2000 as inidcated on manufacturer website : https://www.rndsystems.com/products/streptavidin-hrp_dy998)
Rabbit anti-MEV HRC4 +/- HRP (Genscript, on demand custom production, validated by ELISA)

Validation

All commercialy available antibodies were validated by the manufacturer and the data is available in their corresponding websites, listed above. The additional validation of cell type and species specificity hwas done in Bjornson-Hooper et al (doi: https://doi.org/10.1101/577759)

Eukaryotic cell lines

Policy information about cell lines

Cell line source(s)	Vero cells expressing human SLAM (Vero-hSLAM : ECACC 04091501) , HEK293T (ATCC CRL 3216)
Authentication	The cell lines used were not authenticated.
Mycoplasma contamination	All cell lines were tested for mycopasma on monthly bases and found to be uncontaminated.
Commonly misidentified lines (See ICLAC register)	No commonly misidentified lines were used.

Animals and other organisms

Policy information about studies involving animals; ARRIVE guidelines recommended for reporting animal research

Laboratory animals

10 healthy 3-years old femalle cynomolgus macaques (Bioprim), 52 CD150xIFN a/b Receptor KO mice, 31 males and 21 females (breed at PBES, ENS-Lyon, France). All mices were three to four weeks old (both males and females housed separately). Mice were housed with a light cycle of 13h/11h at 22°C+/- 2°C and 50% +/-10% humidity.

Wild animals

No wild animals were used in this study

Field-collected samples

No field-collected samples were used in this study.

Ethics oversight

Animals were handled in strict accordance with good animal practice as defined by the French national charter on the ethics of animal experimentation and all efforts were made to minimize suffering. Animal work was approved by the Regional ethical committee and French Ministry of High Education and Research under following agreements :
- For the pharmacokinetics and toxicology of HRC4 peptide in monkeys, experiments were done at Cynbiose, Marcy l'Etoile, France, accredited by AAALAC. The protocol was approved under number 16 MESR N° 2016072117544328.
- The experiment using measles virus was performed in BSL2 primate facility at the University of Tours, France and received approval under the agreement reference MESRN N°29992-2021022209579514.
- Experiments with mice were done in PBES, ENS-Lyon, France and approved under number APAFIS 21141-2019042916294753v5.

Note that full information on the approval of the study protocol must also be provided in the manuscript.

Flow Cytometry

Plots

Confirm that:

☒ The axis labels state the marker and fluorochrome used (e.g. CD4-FITC).

☒ The axis scales are clearly visible. Include numbers along axes only for bottom left plot of group (a 'group' is an analysis of identical markers).

☒ All plots are contour plots with outliers or pseudocolor plots.

☒ A numerical value for number of cells or percentage (with statistics) is provided.

Methodology

Sample preparation	The 4ml EDTA-treated blood samples were centrifugated in CPT tubes (Becton Dickinson), the PBMC rings were collected and cells were washed twice in RPMI. Cells intended for RNA extraction were separated and the remaining cells were used for different flow cytometry panels (about 3.10e5 cells each). For flow cytometry, cells were re-suspended in 250 µl PBS 2% FCS and incubated for 40 min on ice with 4µl of each antibody. Cells were then washed twice with PBS 2% FCS, fixed 20 min with 4% paraformaldehyde (methanol free), washed with PBS and analysed on a Miltenyi Macquant 10 flow cytometer.
Instrument	Milteny MACSQuant®10 flow cytometer
Software	FlowJo 10.8 (Bcton Dickinson) and Kaluza 2.1 (Beckman Coulter),
Cell population abundance	Depending on cell isolation yeld, 2.10e5 to 1.5.10e6 total events were acquired leading to a minimum of 10e5 cells in the main gate (FSC/SSC) (10e5 up to 8.10e5 cells). Excluded cells presented cell debris, platelets and few remaining erythrocytes and granulocytes, determined based on their morphology (FSC/SSC).
Gating strategy	In figures 6 and 7 and supplementary S7 cells were first gated for morphology (FSC-A/SSC-A), then for duplet exclusion (FSC-A/FSC-H, then SSC-A/SSC-H). B cell population was determined as CD3- CD20+ cells and percentage of GFP positive B cells was analyzed on a FSC-A /GFP dot plot. Monocytes infection was determined on a FSC-A/GFP dot plot gated on CD3-CD20-. T cells subpopulations infection were determined on FSC-A/GFP dot plot gated on CD20-CD3+CD8- or CD20-CD3+CD8+ cells. In figure 8 cells were first gated for morphology (FSC-A/SSC-A) and then for CD20+/CD27+. Finally, a dot plot CD38 vs IgD gated on CD20+/CD27+ cells was used to quantify CD38+/IgD+ and CD38+/IgD- cells.

☒ Tick this box to confirm that a figure exemplifying the gating strategy is provided in the Supplementary Information.
